# Perceived impacts of medications for opioid use disorder implementation on mental health services and substance use counseling in carceral settings: qualitative findings from 13 Massachusetts jails

**DOI:** 10.1186/s13722-025-00641-3

**Published:** 2025-12-22

**Authors:** Ekaterina Pivovarova, Peter D. Friedmann, Warren Ferguson, Benjamin J. Bovell Ammon, Thomas J. Stopka, Elizabeth A. Evans

**Affiliations:** 1 Department of Family Medicine and Community Health, University of Massachusetts Chan Medical School, Worcester, MA USA; 2 Department of Population and Quantitative Health Sciences, University of Massachusetts Chan Medical School, Worcester, MA USA; 3 Department of Medicine, Baystate Health, Springfield, MA USA; 4 Department of Public Health and Community Medicine, Tufts University School of Medicine, Boston, MA USA; 5 Department of Health Promotion and Policy, School of Public Health and Health Sciences, University of Massachusetts Amherst, Amherst, MA 01003 USA

**Keywords:** Medications for opioid use disorder, Carceral, Jails, Implementation, Mental health services, Substance use counseling, Qualitative

## Abstract

**Background:**

Although research on Medications for Opioid Use Disorder (MOUD) in carceral settings has grown, it has largely focused on the implementation of medication delivery or on substance use outcomes in the community. However, the introduction of new programs or the expansion of treatment services in criminal legal settings can have both direct and indirect consequences on other treatment programs and correctional operations within jails. Mental health and substance use disorders frequently co-occur, and their psychosocial treatment components often overlap. We examined how the implementation of MOUD in all jails across Massachusetts impacted the mental health services operating within the jails and the requirements for substance use counseling alongside MOUD.

**Methods:**

We conducted semi-structured interviews (*n* = 47) and focus groups (*n* = 42) with staff from 13 county jails as part of an implementation of MOUD in jails study. Using deductive and inductive coding, all transcripts were double-coded and analyzed using a modified framework method.

**Results:**

We identified five key themes about the perceived impact of MOUD on mental health and substance use counseling services. First, MOUD implementation was perceived to reduce acute mental health crises, such as risk for suicide, and the demand on mental health services at intake to the facility. Second, staff perceptions about the effectiveness of MOUD as a stand-alone treatment influenced their decisions about the need for and interpretation of substance use counseling requirements. Third, the required components of substance use counseling created a need for additional staff, which exacerbated the existing shortage of mental health staff. Fourth, infrastructure limitations and privacy needs made the delivery of substance use counseling logistically challenging in jail settings. Finally, MOUD implementation increased interdisciplinary collaboration in some jails by requiring medical, mental health, and substance use providers to work together to resolve the needs of incarcerated individuals.

**Conclusions:**

As jails aim to meet regulatory requirements for MOUD, they will need to manage potential staffing shortages, infrastructure constraints, and shifts in the mental health and substance use counseling services. Guidelines for implementing MOUD in carceral settings should also consider the unintended consequences of MOUD on other behavioral health services.

**Supplementary Information:**

The online version contains supplementary material available at 10.1186/s13722-025-00641-3.

## Introduction

Overdose remains the leading cause of death among individuals involved in the criminal legal system [1–3]. In response, an increasing number of carceral facilities have begun offering all forms of medications for opioid use disorder (MOUD) to reduce overdose deaths in this high-risk population [4]. Nationally, approximately 40% of jails offer some MOUD, yet fewer than 13% offer it to all individuals diagnosed with OUD [5]. In Massachusetts, a legislative mandate led to a pilot program providing all FDA-approved MOUD in five county jails, with two additional jails joining voluntarily [6]. Within four years, nearly all Massachusetts jails were offering all types of approved MOUD. Evidence-supported guidelines now inform the implementation of all types of MOUD in correctional settings [7–9]. Despite increasing research on MOUD implementation in carceral settings, the focus has primarily been on the medication delivery process [9–11] or substance use and criminal legal outcomes [12, 13]. However, the introduction of new programs or expansion of treatment services in criminal legal settings can have both intended and unintended consequences on existing programs and correctional operations [14–16]. For example, a California statewide initiative to provide addiction treatment in lieu of incarceration for adults with nonviolent drug-related charges expanded addiction treatment capacity [17] but an unintended consequence included the displacement of patients [18] who were seeking treatment voluntarily [17]. To our knowledge, no study has systematically examined whether and how the implementation of all types of MOUD in jails had impacts on existing jail-based mental health services or the counseling components of agonist-based MOUD treatment.

Co-occurring substance use and mental health disorders are highly prevalent in carceral settings, requiring additional staff and programming to address these complex needs [19–21]. Incarcerated individuals with severe mental health disorders such as schizophrenia and bipolar disorders are more likely to have substance use disorders than the general carceral population, with women being at an especially high risk. For example, one study examining a New York state county jail found that up to 61.3% (*n* = 230) of those receiving all types of MOUD also had a history of mental illness, including mood, anxiety disorder, or neurodevelopmental (i.e., ADHD) disorders [15]. A meta-analysis of 34 studies found a lifetime prevalence of 39.9% for serious mental illness (i.e., psychotic and mood disorders) and substance use disorders [21]. Additional concerns for individuals with co-occuring mental health and substance use disorders include higher risks for rearrest, reincarceration, and harsher disciplinary consequences [20, 22, 23]. Despite this high level of need, jails and prisons often struggle to deliver adequate mental health care, in part due to persistent shortages of qualified professionals [24]. It is unknown how the introduction of MOUD would further impact already overburdened behavioral health services within correctional facilities.

Federal regulations for the provision of substance use counseling alongside agonist-based MOUD vary by MOUD type and may be further supplemented by state-specific counseling requirements. Federal guidelines for methadone providers require that programs provide adequate substance use disorder counseling to each patient as clinically necessary and mutually agreed upon. Federal guidelines for buprenorphine require that prescribers provide access to substance use counseling [25–27]. The effectiveness of substance use counseling requirements with agonist-based MOUD on treatment engagement, retention, and overdose has been equivocal [28]. A meta-analysis of 48 studies found that the adjunctive psychosocial treatment to agonist-based MOUD found largely no difference in substance use outcomes with or without adjunctive treatment [28], with the exception of contingency management treatment. However, a multisite randomized controlled trial of buprenorphine found that individuals with posttraumatic stress disorder who received adjunctive counseling did have significantly and clinically better outcomes than those who did not receive counseling [29]. Carceral-legal staff report that substance use counseling should be provided alongside MOUD, with fewer than 10% of staff believing that MOUD should be prescribed without required substance use counseling [30]. These beliefs are likely to stem, at least in part, from stigmatizing beliefs that agonist-based MOUD substitutes one drug and reflects individuals’ moral failings, in turn creating systemic barriers to treatment, such as additional requirements to receive MOUD [31–34]. Understanding the perceived impact of medication administration on substance use counseling needs and services in jails may inform effective and pragmatic regulations around MOUD in carceral settings.

Further complicating service delivery is the potential overlap between mental health and substance use counseling [35, 36]. A broad range of professionals can deliver substance use counseling. However, alcohol and drug counselors (a common type of provider for substance use counseling) typically focus on substance use and often only require a bachelor’s degree. In contrast, mental health counselors generally address a broader spectrum of conditions and are required to have a master’s degree but may have limited training in substance use treatment [37]. Treatment techniques in mental health and substance use counseling also overlap. For example, motivational interviewing is used in both mental health [38] and substance use counseling [39]. As a result, resource-limited settings, like jails, and those implementing programs with substance use counseling requirements may face particular challenges in determining who should deliver which service and how those services fulfill MOUD counseling requirements.

As part of a five-year study examining the implementation of MOUD in Massachusetts jails, we interviewed jail staff to understand how the availability of agonist-based MOUD in their jails impacted their work and existing services. This study examines how agonist-based MOUD administration is perceived to impact mental health and substance use counseling needs and services.

## Methods

### Participants and recruitment

Semi-structured individual interviews (*n* = 47) and focus groups (*n* = 42) were conducted with jail staff from all 13 of Massachusetts’s county jails. Focus groups ranged in size from 2 to 8 participants and were grouped based on staff roles, expertise, and seniority. In total, 118 individuals participated in focus groups. See Table 1 for descriptive information about who participated in interviews and focus groups (*n* = 172). We used purposive sampling to identify senior and line jail staff who actively participated in and made decisions about the implementation of MOUD in their jail. With the support of senior jail administrators, we targeted key jail staff and shared recruitment materials about the study. Inclusion/exclusion criteria were being 18 years of age or older, working in a participating study jail, and the ability to provide informed consent.

### Procedures and data collection

The Exploration, Preparation, Implementation, and Sustainment framework (EPIS [6, 40]) guided data collection, interview and focus group instrument development, and data coding and analyses. EPIS was selected given its utility in describing the implementation of interventions in criminal legal settings and with substance use disorders [41–43]. One-hour semi-structured interviews were conducted in person at the jails or via Zoom. Semi-structured focus groups of approximately 90 min in duration were conducted in person. Data were collected in two waves. The first, between 2019 and 2020, was for the original seven jails that either voluntarily joined or were mandated by the MA legislative order to implement MOUD treatment. The second, between 2022 and 2023, was for later adopters, focused on eight jails that implemented MOUD later (either voluntarily or in response to a Department of Justice agreement). All interviews and focus groups were conducted in private spaces and audio recorded, transcribed verbatim, and redacted. Interviewees also completed a demographic form. Interviewees did not receive payment due to jail regulations. All participants provided informed consent before participation. The Baystate Health IRB served as the single IRB. See Table 1 for participant demographic information.

### Data coding and analyses

In developing the codebook, study investigators and six masters’ level research coordinators began with a priori codes from the EPIS framework and published research [42]. The codebooks were refined by reviewing three transcripts for conceptual categories and salient subcategories using the constant comparative method [44]. Separate codebooks with similar codes were used for the two data collection waves - early adopters (Wave 1) and later adopters (Wave 2). The early adopters codebook had 16 parent codes and 38 child codes. We used the early adopter codebook to develop the later adopter codebook, which was ultimately pared down to 10 parent categories and 27 child codes. We examined the following codes: (1) additional services related to MOUD but not medication, (2) mental health services and needs, and (3) staff capacity related to hiring and needs. To ensure these codes captured our intended topic, we also conducted a word search for terms “mental,” “mental health,” “behavioral,” and “psych” with findings overlapping with reviewed codes.

Research staff established coding consistency by coding five transcripts altogether. Subsequently, the staff worked in dyads to code each transcript independently and met as a group to review coding applications and resolve discrepancies. Study investigators met coders every two weeks to address questions and review progress. All codes were entered into Dedoose (9.0.17 version) [45]. A modified Framework Method [46] was used to analyze and organize coded segments, with at least two co-authors reviewing each segment to identify salient categories and thematic saturation. Quotes were selected to demonstrate agreed-upon categories.

## Results

Responses are categorized into five key themes of how implementation of MOUD was perceived to impact mental health services and substance use counseling in jail, including: (1) reduced acute mental health needs of incarcerated persons; (2) substance use counseling requirements were shaped by perceptions of MOUD; (3) exacerbated staffing shortages for mental health and substance use counseling roles; (4) created challenges in adapting substance use counseling requirements to jail contexts; and (5) enhanced interdisciplinary collaboration.

### Reduced acute mental health needs of incarcerated individuals during withdrawal

Interviewees consistently described that one of the most notable impacts of MOUD on mental health services was a reduction in the acute mental health needs of incarcerated individuals. Before MOUD was offered in jails, individuals entering the facility who were using opioids or receiving methadone or buprenorphine medications in the community experienced severe withdrawal symptoms upon incarceration due to forced withdrawal. Jail staff described how managing these symptoms was time-, space-, cost-, and staff-intensive, burdening already limited jail resources. Staff had to house individuals experiencing acute withdrawal on medical units, continuously monitor them for safety due to suicide risk, and give medications to address behavioral and mood instability, often referred to as “comfort medications”. When MOUD became accessible in the jails, individuals no longer experienced withdrawal and the staff required fewer resources to manage them.

One correctional officer (ID090915) explained that the traditional withdrawal management protocol, where “we give them several medications for them to detox,” is “unbearable” for incarcerated persons, requiring close mental health observation to ensure the individual’s safety. A case manager observed a reduction in need for mental health services immediately upon incarceration since MOUD was implemented.

I definitely think [we are] seeing a lot less of those problems in the beginning, because…you’d have more maybe suicidal attempts, or more like mental health calls and then mental health is…running around and then you’re having to transport people to

medical who are just in such pain withdrawing [and] that’s why they’re having these crises…This whole big group [of incarcerated persons] now is kind of just not a problem, because they’re more comfortable considering being here (ID 090610)

One nursing staff member also described that starting individuals on MOUD has helped to isolate mental health symptoms from addiction and address the need for dual diagnosis treatment.

MAT has really allowed us…to focus on serious mental illness…For example, we have a pretty well-known detainee that unfortunately comes in a couple of times a year, but every time he comes in…you’re getting a call from the officer, ‘should we put him on a watch now?…He’s gonna hurt himself.” And now…all I need to do is call [MOUD staff] and say, “Can you start the Suboxone?” And, it’s just immediately he’s just like a new man…Just knowing that we have that option to treat someone the right way rather than before…treat them with so many other band aids (ID 070202)

Similarly, a senior administrator observed that MOUD also reduced the work burden for correctional staff, as it reduces behavioral health needs.

Corrections officers who recognize that continuing patients on this treatment, not only is it the right thing to do for the patient, but it’s the right thing to do for the correctional environment…I’ve had unsolicited comments from the team where they’ll say, ‘wow, these patients are so much easier to manage. There’s so many less behavioral issues since we’ve allowed them to continue their medication’…Yes, that is the primary reason for you guys to support this. It makes your job easier. It makes them healthier, and it makes your job easier (ID120304)

### Interpretation of substance use counseling requirements shaped by perceptions of MOUD

Jails differed in their requirements for participation in substance use counseling. Some facilities mandated daily or weekly participation in group counseling, individual therapy, or mutual support groups (e.g., AA), while others required monthly patient check-ins with their counselor. All staff reported that non-compliance with psychosocial treatment alone did not result in discontinuation of MOUD. Instead, staff described that “we want to motivate them to come to groups” (ID110203) and to encourage the non-MOUD components of MOUD programs. In part, the extent of substance use counseling requirements of MOUD reflected jail leadership’s beliefs about the effectiveness of MOUD without counseling. One senior jail administrator reported,

I’m not a big believer in the [MOUD] program as much as I am in the counseling…I’m not a fan of the drug side of it. I’m a big believer in the counseling side of it…I wanted to make sure whatever we did [in implementing MOUD], we had a strong counseling component with it. (ID 120405)

Some staff reported stigmatizing beliefs and misconceptions about MOUD, emphasizing behavioral and attitude changes as primary drivers of recovery, and not the medications per se.

It’s not just medication. It’s really changing their behavior because what happens is they go out into the community and…they all get on the same medications. It’s Suboxone, but it’s Xanax, it’s Gabapentin, and it’s Adderall. That’s the cocktail that they all take and…if they go back to those medications, the chance for them going into any kind of recovery, in my mind, it’s just nil because all they’re doing is just substitute…If we don’t change that mindset and get them to say, “No…I want a recovery. I don’t want a substitute”…the outcome is not going to be what we want…So that’s more of the conversations I have when I try to get them thinking about different things. (Senior Administrator and Physician, ID 060407)

Other staff perceived MOUD as effective on its own and described tailoring substance use counseling to the individual needs of the incarcerated person. One staff explained,

[Staff member] lets them know about what’s required for the [MOUD] program…Taking the medication the way it is prescribed…Following certain rules and, clinicians, we try to meet with each patient twice a month. One group and one individual…We try to meet people where they’re at…We’re not trying to force counseling if that’s not something they want, but we check in on them. So, it’s kind of…helping them develop one of the skills they want to develop, kinda like a harm reduction way. (MOUD Director, ID 090101)

### Exacerbated staffing shortages for mental health and substance use counseling roles

Although staff described how the implementation of MOUD reduced the need for staff to manage acute withdrawal symptoms, it also required additional staffing to provide intake evaluations as well as group and individual substance use counseling. However, this has also meant that existing mental health services have had to be expanded to incorporate substance use counseling. This was further exacerbated by the overlap in the types of services that mental health and substance use disorder specific counselors could offer. One clinical supervisor explained how they did mental health assessments for MOUD intake, which may have identified additional people with co-occurring mental health and substance use conditions,

We do intake assessments like behavioral health intake assessments on everybody that comes in that’s on MOUD, whether they are sentenced [or] pre-trial…We’re having to do all of those assessments…It also means they’re all in [therapy] treatment…it’s adding groups and [we are] making sure there are enough groups for everybody to fit in…The volume of groups that we’ve got to do has had to increase. (Clinical Supervisor, ID 020306)

Interviewees described experiencing substantial staffing shortages for existing mental health services that were further exacerbated by additional needs for substance use counseling. Staff reported that it was especially challenging to hire mental health practitioners to work in carceral settings because of the already existing shortages for these roles in the community.

[PROVIDER Organization] has trouble bringing staff [to the jail]…people don’t want to work in jail. People don’t go through all this college, come sit and work in jail, honestly, they don’t. A lot of people [who start] here, don’t stay for long. (Correctional Officer, ID 100305)

Some interviewees believed that hiring for medical and correctional positions was prioritized over substance use counseling and case management roles. One MOUD counseling provider explained:

We have…two dosing nurses, a doctor, a nursing manager, and a nurse practitioner on the medical side. We have three different security [officers]. And on the clinical side, there’s really just me there five days a week. My clinical supervisor is not really a clinical supervisor, and the part-time clinician is there two days a week…When I brought up the need to have a regular clinician assigned other than myself, ‘it’s not in the matrix’ is what I was told. I was told the same thing around having a program director. (MOUD counseling provider, ID 100407)

The MOUD provider went on to describe lack of professional expertise in addictions treatment to support existing staff in their roles and a rigid chain of command.

…Right now, we have my clinical supervisor, who is really the mental health director, who’s not really proficient with MAT…What happens is I’m coming up with all the questions, and she doesn’t know the answer, and she goes above someone else, and it gets funneled back down…If I go above her to try to get an answer directly, then it’s, “Don’t do that.” It’s so hierarchical. Because there are many eyes and ears in the [MOUD] program, because it’s so new, and walking the political land is the most difficult part for me. (MOUD counseling provider, ID 100407)

### Created challenges in adapting substance use counseling requirements to jail contexts

Interviewees described the complexities of providing substance use counseling for individuals on MOUD within the confines of a highly structured and security-focused environment. While jails developed the medical and security infrastructure to deliver medication, private spaces for “talk therapy,” whether in individual or group formats, were often lacking. Counseling staff reported frequently doing therapy in open spaces with other incarcerated persons and staff within earshot. Interviewees described that even when private space was available, it was difficult to establish the rapport necessary for counseling in jail environments.

…It’s a very very difficult environment to open up…[for] a client. And sometimes when I’m asking them…it’s very very uncomfortable for them, because they don’t want to open that up and then be walked back to their unit…Some of them are very very open to talking about it, but some of them I think skim over it. (MOUD Provider, ID 100407)

Interviewees also noted that even when there were fewer expectations of privacy, such as in groups, incarcerated individuals were hesitant to participate with correctional staff present.

…The groups, especially in the beginning, when it first started…they’re [incarcerated individuals] not very comfortable talking with any kind of security around because they’re guarded. (Senior Correctional Officer, ID 091118)

One interviewee described how incarcerated individuals were less willing to disclose during substance use counseling because of concerns that they would be perceived as mentally ill and require psychiatric interventions.

…Sometimes, I think people are less likely to ask for support from the jail staff because there’s fear of, “Am I going to get on suicide watch?…What is going to happen? Is there going to be some stigmatization based on what I’m reporting, and will I be treated differently?” So, it’s a complicated thing to navigate for sure. (MOUD Director, ID 120203)

As a result, staff explained that the substance use counseling format may have differed from that provided in the community, focusing more on “coping mechanisms, not really [long-term] treatment.” To accommodate this, one jail adjusted by offering more peer-run groups.

We’ve been pretty successful because it’s a peer, most of our groups are peer-based…[We] always let them know, we only facilitate the group. Many times, we have had topics, self-love might be the topic. We usually go around and ask people how they [are] doing. Maybe one person might have talked to that significant other. They’re upset, different things like that, quickly, the meeting goes to that person. So that might be the conversation for the day, where other people can identify to help that individual…Even if we have a topic, sometimes, a lot of times it don’t go as planned. (Program Coordinator, ID 110203)

Interviewees also explained that lockdowns and movement restrictions interrupted sessions. And, while medication was always distributed during or immediately after lockdowns, counseling sessions were not always rescheduled. The logistical limitations described by interviewees are common to the provision of mental health counseling services in carceral settings as well, but these may have been more pronounced given the rapid increase of individuals receiving those services (those on MOUD) and staff training in the provision of substance use counseling.

### Enhanced interdisciplinary collaboration

Implementation of MOUD was a considerable change to the existing jail ecosystems, where medical and mental health services typically operated in silos. The introduction of a third treatment service, which spanned medication and counseling, led some jails to improve their interdisciplinary and inter-service collaboration. Additionally, cross-utilization of some staff from mental health services to provide MOUD counseling helped streamline communication. One senior correctional administrator explained:

We were always at case management meetings, but this particular area of prescribing psychotropic medication or MOUD was kind of the catalyst that potentially aided greater growth and greater collaboration. (Senior Correctional Administrator, ID 020823)

One jail purposefully addressed the disconnect between services by integrating mental health and substance use counseling services within broader medical and correctional services.

We meet every single week as an integrated behavioral health and medical group. That is a real change. It took us a while to get there. They were silos, behavioral health lived in one, mental and the psychologist lived in one, and medical lived in one. And now we really are all blended together and with security too, so at this behavioral health table, security, clinical case workers, social workers, medical, the nurses, me and we talk about the patients and we talk about their mental health and we talk about the case workers and the correctional officers give feedback, they say, “this guy looks really sedated during the day at three in the afternoon and why is that?” (Medical Director, ID 020722)

Staff in other jails, however, observed that the silos remained, and the disconnect between mental health, addiction, and medical services remained, including the use of separate electronic platforms and the provision of services by different contractors.

I feel like everything here is very boxed off and sectioned on its own…that becomes challenging, just communication. A lot of the time, I don’t know how they’re doing with their substance abuse stuff, which does play a role a lot in just their mental health overall. (Medical Provider, ID110305)

## Discussion

From a large statewide sample of jail staff, we found that the implementation of MOUD in jails impacted not only addiction-related services but also broader behavioral health services provided in the jail. Staff described both system-wide benefits and challenges associated with MOUD implementation. Benefits included a reduction in acute mental health needs of individuals experiencing withdrawal and enhanced interdisciplinary collaboration. In particular, interviewees described how the implementation of MOUD, and thereby the avoidance of acute withdrawal symptoms, potentially reduced suicide attempts and eased the management of behavioral health needs of individuals entering the facility. However, staff also reported challenges from the increased demand for staff to deliver required substance use counseling, alongside MOUD, and difficulties in providing substance use counseling in the jail context. Notably, staff perceptions of the effectiveness of MOUD as a stand-alone treatment influenced how requirements for substance use counseling were interpreted and implemented. These perceptions also shaped the perceived need for specialized staff and infrastructure to support counseling alongside MOUD.

Our findings also reveal how some adaptations had a positive overall system effect, such as the integration of substance use, behavioral health, and medical services. This aligns with community research findings that the provision of integrated mental health and substance use disorders treatment is associated with better health and legal outcomes [47, 48] than each treatment alone. Extending effective practices about integrated care in the community to correctional settings may lead to overall improved care of individuals who may cycle in and out of jails. However, other reported adaptations to MOUD implementation, such as having staff conduct substance use counseling without the requisite expertise, may be less effective. This may be especially concerning in carceral settings, where healthcare staff may feel limited clinical decision-making autonomy and be concerned about ethical compromises [49].

We also found that the frequency and nature of substance use counseling differed across the jails and were impacted by staff perceptions about the need for substance use counseling treatment alongside medication. In some cases, these perceptions may have reflected stigmatizing beliefs that do not consider remission on MOUD to be recovery and thereby resulted in reduced access to MOUD [34]. They also could have stemmed from beliefs about the need to individualize treatment and staff recognition of the high rates of co-occurring mental health and substance use disorders. Clear, consistent, and empirically driven guidelines and policy are necessary, not only about whether substance use counseling should be provided alongside MOUD, but also about the frequency, type, and target population that should receive this service.

In examining the impact of MOUD on existing mental health services within the jail, we found that MOUD reduced immediate staffing and space needs by addressing acute mental health symptoms of individuals experiencing withdrawal symptoms. However, these resource needs appeared to shift to later stages of an individual’s incarceration, to accommodate the required substance use counseling. These findings align with prior research, which reported that staffing concerns were a major barrier to initiating MOUD services [5]. Jails will also need to consider the logistical challenges of establishing confidential spaces for substance use counseling, beyond those typically used for mental health services.

Using the EPIS framework, our findings indicate that implementation was impacted by the interactions between the inner context (i.e., existing treatment services, staffing expertise, logistical barriers) and outer context (i.e., state and federal requirements about counseling services, incarcerated persons’ willingness to participate in counseling, overall staffing shortages). In part, the imprecision of MOUD counseling requirements may have led the jails to interpret the innovation (i.e., MOUD) differently, where, in some cases, stigmatizing beliefs guided whether and how substance use counseling should be provided with MOUD. Interdisciplinary collaboration served as a bridging factor to reducing barriers to MOUD and duplication of treatment services.

The introduction of any innovation, such as MOUD, produces ripple effects that impact multiple services and systems within the facility. Accordingly, our work suggests that planning for MOUD implementation in jails, should specifically consider staffing and infrastructure necessary to provided adjunctive substance use counseling while also accounting for potential overlap with mental health services. Prior studies of MOUD implementation costs in jails developed budget impact tools [50, 51] and examined different models specific to medication delivery only. Future research should examine ancillary costs to the jail, inclusive of the potential impact of MOUD on other health services within the facility. Similarly, it may be helpful to expand existing guidelines for MOUD implementation in carceral settings to specifically include preparation for potential changes to the overall care of incarcerated individuals receiving MOUD.

A strength of this study is its in-depth examination of the impact of implementing MOUD on counseling and existing mental health services across a statewide sample of jails. Nonetheless, its findings should be interpreted within existing limitations. First, we did not directly recruit mental health staff (e.g., psychiatry, psychology, social work) unless they were involved in the MOUD program. It may be that those involved in the implementation perceived more relevance between MOUD and mental health services than staff whose roles do not overlap with any MOUD services. Second, we did not directly ask about the impact of MOUD on incarcerated persons’ mental health, those with co-occuring conditions, or on mental health services only; instead, we explored the impact of MOUD broadly on jail operations. Third, interviews occurred primarily during the early implementation of MOUD, and some challenges may have been addressed with time. Nevertheless, planning for the impact of MOUD on counseling and mental health services will be critical for other carceral agencies considering its provision. Additionally, our study focused on jails in MA, which may limit transferability. MA has the lowest incarceration rate in the country and has relatively robust substance use and mental health resources compared to other states. These issues may be even more pronounced in states with fewer resources. Finally, some of our data were collected during the COVID-19 pandemic. Accordingly, some of our findings may reflect disruptions to carceral and health protocols at that time.

## Conclusion

Our findings highlight the impact of MOUD implementation on the mental health needs of, and services for incarcerated patients and substance use counseling. As jails implement MOUD, they will need to manage potential shortages of counseling staff, infrastructure constraints, and shifts in the mental health needs of incarcerated individuals. Guidelines for the implementation of MOUD in carceral settings should also consider its unintended consequences on behavioral and other health services.

**Table 1:** Demographics of staff participating (*n* = 172) in focus groups and interviews

Characteristic	*N* (%)
**Age**, mean (SD)*	44.1 (11.4)
**Female**, n (%)*	99 (57.9%)
**Ethnicity**, n (%)*	
Non-Hispanic	162 (94.7%)
**Race**, n (%)**	
Asian	5 (3.0%)
Black or African American	11 (6.5%)
White	153 (90.5%)
Other (not specified)	3 (1.8%)
**Highest Level of Education**, n (%)*	
High school diploma	10 (5.8%)
Some college, but not degree	18 (10.5%)
Associate’s degree	23 (13.5%)
Bachelor’s degree	50 (29.2%)
Master’s degree	51 (29.8%)
Doctoral degree or equivalent	16 (9.4%)
Other (not specified)	3 (1.8%)
**Current Position**, n (%)**	
Case worker for incarcerated individuals (e.g., re-entry planner)	33 (19.5%)
Caseworker for recently released individuals (e.g., community navigator)	5 (3.0%)
Mental Health and/or addiction counselor	22 (13.0%)
Administrative staff (e.g., superintendent, administrator, senior director, etc.)	32 (18.9%)
Medical (e.g., physician, nurse, etc.)	42 (24.9%)
Correctional/Security	22 (13.0%)
Other (not specified)	13 (7.7%)
**Years working in current position**, n (%)*	
<1 year	33 (19.3%)
1–3 years	60 (35.1%)
4–9 years	35 (20.5%)
≥10 years	43 (25.1%)
**Years working for current jail**, n (%)*	
<1 year	24 (14.0%)
1–3 years	39 (22.8%)
4–9 years	37 (21.6%)
≥10 years	71 (41.5%)
**Years offering MOUD at current jail**, n (%)**	
<1 year	42 (24.9%)
1–3 years	80 (47.3%)
4–9 years	42 (24.9%)
≥10 years	5 (3.0%)

Note: *Missing = 1, ** Missing = 3. Abbreviations: MOUD = Medications for opioid use disorder; SD = Standard Deviation

## Supplementary Information

Below is the link to the electronic supplementary material.

Supplementary Material 1

## Data Availability

No datasets were generated or analysed during the current study.
