# Supplementary material for: Perceived impacts of medications for opioid use disorder implementation on mental health services and substance use counseling in carceral settings: qualitative findings from 13 Massachusetts jails
Source: Addict Sci Clin Pract. 2025 Dec 22;21:11. doi: 10.1186/s13722-025-00641-3 (PMC12825220; doi:10.1186/s13722-025-00641-3)
Supplement: Supplementary file 1 — Supplementary Material 1 [file 13722_2025_641_MOESM1_ESM.docx]

Appendix. COREQ (COnsolidated criteria for REporting Qualitative research) Checklist

| **Topic** | **Item No.** | **Guide Questions/Description** | **Details** |
| --- | --- | --- | --- |
| **Domain 1: Research team and reflexivity** | | | |
| *Personal characteristics* | | | |
| Interviewer/facilitator | 1 | Which author/s conducted the interview or focus group? | EP, EE, PF, TS, WF |
| Credential | 2 | What were the researcher’s credentials? E.g., PhD, MD | PhD, MD, MPH, MHS |
| Occupation | 3 | What was their occupation at the time of the study? | Psychologist, Addiction Medicine Physician, Epidemiologist, Family Medicine Physician, and Public Health Researcher |
| Gender | 4 | Was the researcher male or female? | Females and males |
| Experience and training | 5 | What experience or training did the researcher have? | Interviewers had extensive training and history of conducting qualitative research interviews, years of substance use and public health research |
| *Relationship with participants* | | | |
| Relationship established | 6 | Was a relationship established prior to study commencement? | Interviewers introduced themselves as research team members to study implementation of MOUD in jails. |
| Participant knowledge of the interviewer | 7 | What did the participants know about the researcher? e.g., personal goals, reasons for doing the research | Interviewers described their research roles, academic affiliations, and purpose for conducting research |
| Interviewer characteristics | 8 | What characteristics were reported about the interviewer/facilitator? e.g., Bias, assumptions, reasons and interests in the research topic | The education levels, sex, and disciplines, of the interviewers are reported in the manuscript. |
| **Domain 2: Study design** | | | |
| *Theoretical framework* | | | |
| Methodological orientation and Theory | 9 | What methodological orientation was stated to underpin the study? e.g., grounded theory, discourse analysis, ethnography, phenomenology, content analysis | Exploration, Preparation, Implementation and Sustainment (EPIS) framework drove instrument development, data collection, and data analyses.  Adapted framework analysis method was used to analyze data. |
| *Participant selection* | | | |
| Sampling | 10 | How were participants selected? e.g., purposive, convenience, consecutive, snowball | Purposive sampling of key jail staff involved in MOUD implementation. |
| Method of approach | 11 | How were participants approached? e.g. face-to-face, telephone, mail, email | Participants were recruited via email and in person. |
| Sample size | 12 | How many participants were in the study? | 172 |
| Non-participation | 13 | How many people refused to participate or dropped out? Reasons? | All individuals that responded to emails about participating enrolled in the study. |
| *Setting* | | | |
| Setting of data collection | 14 | Where was the data collected? e.g., home, clinic, workplace | Jail |
| Presence of nonparticipants | 15 | Was anyone else present besides the participants and researchers? | No |
| Description of sample | 16 | What are the important characteristics of the sample? e.g., demographic data, date | See table 1 |
| *Data collection* | | | |
| Interview guide | 17 | Were questions, prompts, guides provided by the authors? Was it pilot tested? | Questions were developed by the authors and pilot tested with jail staff. |
| Repeat interviews | 18 | Were repeat interviews carried out? If yes, how many? | No |
| Audio/visual recording | 19 | Did the research use audio or visual recording to collect the data? | Audio recordings |
| Field notes | 20 | Were field notes made during and/or after the interview or focus group? | Notes were taken by interviewers |
| Duration | 21 | What was the duration of the interviews or focus group? | Interviews were ~ 60 minutes and groups were ~ 90 minutes. |
| Data saturation | 22 | Was data saturation discussed? | Yes |
| Transcripts returned | 23 | Were transcripts returned to participants for comment and/or correction? | The transcripts were not returned to participants for comment and/or correction. |
| **Domain 3: analysis and findings** | | | |
| *Data analysis* |  |  |  |
| Number of data coders | 24 | How many data coders coded the data? | 6 coders (3 dyads) |
| Description of the coding tree | 25 | Did authors provide a description of the coding tree? | Codes were refined using open coding and constant comparative methods, resulting in a codebook with 23 parent codes and 32 child codes. |
| Derivation of themes | 26 | Were themes identified in advance or derived from the data? | Emergent themes using deductive and inductive approaches. |
| Software | 27 | What software, if applicable, was used to manage the data? | Dedoose v9 (Los Angeles, CA) |
| Participant checking | 28 | Did participants provide feedback on the findings? | Draft of the manuscript was shared with jails partners for review and feedback. |
| *Reporting* | | | |
| Quotations presented | 29 | Were participant quotations presented to illustrate the themes/findings? Was each quotation identified? e.g., participant number | Yes, each quote has an ID and interviewee role |
| Data and findings consistent | 30 | Was there consistency between the data presented and the findings? | Yes |
| Clarity of major themes | 31 | Were major themes clearly presented in the findings? | Yes |
| Clarity of minor themes | 32 | Is there a description of diverse cases or discussion of minor themes? | Yes |

Developed from: Tong A, Sainsbury P, Craig J. Consolidated criteria for reporting qualitative research (COREQ): a 32-item checklist for interviews and focus groups. International Journal for Quality in Health Care. 2007. Volume 19, Number 6: pp. 349 – 357.
